# Development and Internal Validation of the Soluble ST2, Age, and Estimated Glomerular Filtration Rate–Heart Failure Score for Predicting 30-Day Major Adverse Cardiovascular Events After Heart Failure Hospitalization in a Two-Center Vietnamese Cohort: Prospective Cohort Study

**DOI:** 10.2196/97879

**Published:** 2026-07-29

**Authors:** An Viet Tran, Nguyen Khoi Quoc La, Van Anh Thi Phan, Bao The Nguyen, Hoa Thai Nguyen, Nhung Hong Thi Thai, Tho Anh Kieu Pham

**Affiliations:** 1 Can Tho University of Medicine and Pharmacy, 179 Nguyen Van Cu street, Tan An, Can Tho, 90000, Vietnam, 84 907250077; 2 Thong Nhat Dong Nai General Hospital, Dong Nai, Vietnam; 3 University of Medicine and Pharmacy, Hue University, Hue, Vietnam

**Keywords:** risk score, predictive model, major adverse cardiovascular events, MACE, soluble suppression of tumorigenicity 2, sST2, age, estimated glomerular filtration rate, heart failure, soluble ST2, age, and estimated glomerular filtration rate–heart failure, SAGE-HF score

## Abstract

**Background:**

Patients hospitalized for heart failure (HF) face a high-risk early postdischarge period.

**Objective:**

We aimed to develop and internally validate the soluble ST2, age, and estimated glomerular filtration rate–heart failure (SAGE-HF) score, a simplified risk model incorporating soluble suppression of tumorigenicity 2 (sST2), age, and renal function, for predicting 30-day postdischarge major adverse cardiovascular events (MACE).

**Methods:**

This two-center prospective cohort study included 218 patients hospitalized with acute decompensated heart failure (ADHF) and/or heart failure with reduced ejection fraction (HFrEF) and 59 non-HF controls in Vietnam (July 2025-January 2026). Serum sST2 concentrations were measured at admission using enzyme-linked immunosorbent assay and compared between the ADHF/HFrEF cohort and controls, with adjustment for clinical covariates. Among the ADHF/HFrEF cohort, the primary endpoint was 30-day MACE, defined as a composite of all-cause death or HF rehospitalization. Candidate predictors were considered based on clinical relevance, biological plausibility, and exploratory univariable associations, and prespecified parsimonious logistic regression models were evaluated using discrimination (area under the receiver operating characteristic curve [AUC]), calibration, Brier score, and Akaike information criterion. Internal validation was performed using 1000 bootstrap resamples, and model robustness was assessed using Firth penalized logistic regression. The SAGE-HF score was derived from the final model. All data were analyzed using the R environment (version 4.5.5; R Foundation for Statistical Computing).

**Results:**

Among 277 participants, sST2 concentrations were significantly higher in patients with HF than in non-HF controls after adjustment. Among 218 patients, 47 (21.6%) experienced 30-day MACE. In multivariable analysis, age (odds ratio [OR] 1.04 per year, 95% CI 1.01‐1.07; *P*=.02), sST2 (OR 1.06 per ng/mL, 95% CI 1.01‐1.11; *P*=.01), and estimated glomerular filtration rate (OR 0.98 per unit, 95% CI 0.97‐1.00; *P*=.02) were independently associated with MACE. The final model demonstrated acceptable discrimination (AUC 0.741, 95% CI 0.664‐0.819) and good calibration, with stable performance after bootstrap validation (corrected AUC 0.725). Firth analysis yielded consistent results. The SAGE-HF score showed progressive risk stratification, with predicted 30-day MACE ranging from 7.0% to 60.1% and maintained acceptable discrimination and calibration.

**Conclusions:**

The SAGE-HF score, incorporating age, sST2, and estimated glomerular filtration rate, demonstrated acceptable performance for predicting 30-day MACE after HF hospitalization and may support early risk stratification, pending external validation.

## Introduction

Heart failure (HF) is a major and growing global health problem, with substantial variation in epidemiology across regions and persistent gaps in data from developing countries [1,2]. After hospitalization for acute decompensation, patients enter a high-risk early postdischarge interval, often termed a “vulnerable phase,” during which adverse outcomes—including rehospitalization and death—cluster soon after discharge [3]. Indeed, approximately 25% of patients hospitalized with HF are readmitted within 30 days of discharge, and mortality during this period can approach 10% [4,5]. These early events are not only clinically important but also central to health-system quality initiatives and transitional-care pathways, motivating stronger tools for early postdischarge risk identification.

Despite extensive effort, predicting early postdischarge events in HF remains challenging: readmissions arise from heterogeneous mechanisms and diagnoses, and many existing models show only modest performance or limited portability across settings [4,6]. Current guidelines emphasize guideline-directed medical therapy, careful discharge planning, and timely follow-up, but they do not provide a universally adopted, low-burden, biomarker-enhanced tool specifically optimized for very short-term postdischarge HF outcomes [7]. Identifying whether a small set of routinely available predictors, together with a biologically informative biomarker, can improve short-term risk stratification remains a practical clinical need.

Soluble suppression of tumorigenicity 2 (sST2), part of the interleukin (IL)-33/ST2 axis, is mechanistically linked to myocardial stress, inflammation, fibrosis, and remodeling; circulating sST2 acts as a decoy receptor that neutralizes IL-33 signaling [8]. In acute and chronic HF, sST2 has repeatedly demonstrated prognostic value for mortality and HF hospitalization, including additive value beyond natriuretic peptides in several studies [9-12]. However, clinically usable short-term models that incorporate sST2 remain relatively limited, especially in Southeast Asian settings. Therefore, we conducted a two-center prospective cohort study to develop and internally validate a parsimonious model and simplified point score incorporating admission sST2, namely the soluble ST2, age, and estimated glomerular filtration rate–heart failure (SAGE-HF) score, to predict 30-day postdischarge major adverse cardiovascular events (MACE) after HF hospitalization.

## Methods

### Study Design and Population

This prospective observational study was conducted at Thong Nhat Dong Nai General Hospital and Can Tho Central General Hospital between July 2025 and January 2026. The study population comprised patients hospitalized with acute decompensated heart failure (ADHF) and/or heart failure with reduced ejection fraction (HFrEF) who met the prespecified eligibility criteria. Patients were eligible if they were admitted with ADHF regardless of left ventricular ejection fraction (LVEF) phenotype and/or had HFrEF documented during the index hospitalization. Underlying HF was diagnosed by the treating cardiologists based on compatible symptoms and signs, echocardiographic assessment, and routine clinical investigations during the index hospitalization. ADHF was recorded when patients were admitted because of new or worsening symptoms and signs of HF requiring inpatient treatment. LVEF was assessed by transthoracic echocardiography during the index hospitalization and was used to classify HF phenotype as HFrEF, heart failure with mildly reduced ejection fraction (HFmrEF), or heart failure with preserved ejection fraction (HFpEF). For this study, HFrEF was defined as LVEF ≤40%, HFmrEF as LVEF 41% to 49%, and HFpEF as LVEF ≥50%. Patients with HFmrEF and HFpEF were included only if they were hospitalized with ADHF during the index hospitalization. Therefore, ADHF and HFrEF were not considered mutually exclusive categories. ADHF was considered a clinical presentation, whereas LVEF phenotype was considered an echocardiographic classification. Patients with end-stage chronic kidney disease (estimated glomerular filtration rate [eGFR]<15 mL/min/1.73 m²) or receiving dialysis, those with conditions known to affect sST2 levels, including systemic diseases such as lupus, rheumatoid arthritis, and chronic obstructive pulmonary disease, as well as those lost to follow-up, were excluded. Hospital admission and discharge decisions were made by the treating physicians according to routine clinical practice, and patients were discharged when they were considered clinically stable and suitable for outpatient follow-up. In addition, a non-HF comparison group was recruited for exploratory comparison of baseline characteristics and serum sST2 concentrations. These participants had no known history of HF based on medical record review and clinical history. They underwent clinical assessment to exclude HF, with routine electrocardiogram assessment as part of hospital evaluation. The absence of HF was further supported by N-terminal pro-B-type natriuretic peptide testing and transthoracic echocardiography. A total of 218 patients with ADHF and/or HFrEF and 59 non-HF controls were included in the final analysis.

### Ethical Considerations

The study protocol was reviewed and approved by the Biomedical Research Ethics Committee of Can Tho University of Medicine and Pharmacy, Vietnam, for both participating centers. Ethical approval was granted for the study conducted at Can Tho Central General Hospital under approval number 25.326.HV/PCT-HĐĐĐ, dated June 30, 2025, and for the study conducted at Thong Nhat Dong Nai General Hospital under approval number 25.274.HV/PCT-HĐĐĐ, dated June 30, 2025. The study was conducted in accordance with the ethical principles for biomedical research involving human participants. Written informed consent was obtained from all participants before enrollment. All patient data were coded and kept confidential, and the results were reported only in aggregate form without identifying individual participants. No financial compensation or payment was provided to participants. The manuscript and supplementary materials do not contain any identifiable individual participant images or personal information.

### Outcomes Assessment

The primary outcome was the 30-day postdischarge MACE. For the main outcome analysis, MACE was defined as a patient-level composite end point of all-cause death or rehospitalization for HF during follow-up [13]. During the 30-day postdischarge period, outcome data were initially ascertained by an independent assessor through telephone contact with patients or their family members. This assessor was not involved in sST2 measurement, statistical analysis, or model development and was not provided with baseline sST2 results. Reported events were subsequently verified through a review of hospital and electronic medical records. Rehospitalization for HF was defined as hospital admission due to worsening symptoms or signs of HF requiring inpatient treatment. Deaths during follow-up were identified through telephone follow-up and verified using available medical records whenever possible. Patients were subsequently classified into the MACE and non-MACE groups according to the occurrence of the composite end point within 30 days after discharge. Each patient contributed only once to the primary MACE end point. If both HF rehospitalization and death occurred within 30 days, the patient was counted once in the composite end point and was not double-counted.

### sST2 Concentration Measurements

Serum sST2 concentration was measured using the Elabscience Human sST2 (Soluble ST2) enzyme-linked immunosorbent assay (ELISA) Kit (catalog number E-EL-H6082), a sandwich ELISA assay intended for research use, in accordance with the manufacturer’s instructions. According to the manufacturer’s documentation, the assay sensitivity was 0.19 ng/mL, and the detection range was 0.31‐20 ng/mL. Manufacturer-reported intra-assay coefficients of variation were 4.43%, 4.36%, and 6.31% across low, medium, and high concentration samples, respectively, and inter-assay coefficients of variation were 8.22%, 6.76%, and 6.58%, respectively. Blood samples were collected at hospital admission during the index hospitalization for patients with HF and at enrollment for non-HF comparison participants. Serum samples were processed according to routine laboratory procedures and the manufacturer’s instructions. Samples with optical density values exceeding the upper limit of the standard curve were not extrapolated. Instead, these samples were initially diluted 1:2 using the kit-provided Reference Standard & Sample Diluent and reassayed. If the measured concentration remained above the standard curve range, additional serial dilution was performed as needed until the measured concentration fell within the quantifiable range. Final concentrations were calculated by multiplying the measured concentration by the corresponding total dilution factor. Samples and standards were analyzed in duplicate, optical density was measured at 450±2 nm, and sST2 concentrations were calculated from a 4-parameter logistic standard curve. sST2 values were recorded in nanograms per milliliter and analyzed as a continuous variable to evaluate their association with 30-day postdischarge MACE.

### Other Variables

Baseline variables were collected at admission and included demographic characteristics, clinical profile, medical history, and laboratory parameters relevant to HF prognosis. Age was recorded in years, and BMI was calculated as weight in kilograms divided by height in meters squared. Functional status was assessed according to the New York Heart Association classification [7]. Smoking was defined as a patient who had smoked at least 100 cigarettes during their lifetime and had smoked within 30 days before hospitalization [14]. Alcohol consumption was defined as any self-reported intake of alcoholic beverages, including beer, wine, or spirits, during the 12 months before hospitalization [15]. Comorbidities and clinical history, including hypertension, diabetes mellitus, dyslipidemia, prior myocardial infarction, stroke, atrial fibrillation, smoking, and alcohol use, were obtained from medical records and direct patient interview. LVEF was assessed by transthoracic echocardiography during the index hospitalization, preferentially using the biplane Simpson’s method when image quality was adequate [16]. Serum sodium, urea, and creatinine were measured using routine biochemical assays at admission. eGFR was calculated from serum creatinine using the Chronic Kidney Disease Epidemiology Collaboration equation [17].

### Data Analysis

All statistical analyses were performed in R (R Foundation for Statistical Computing). Data management and variable transformation were conducted primarily with dplyr, while tabular regression outputs were organized with broom and tibble. Continuous variables were summarized as mean (SD) or median (IQR), as appropriate, and categorical variables as frequencies and percentages. Comparisons between patients with HF and controls were performed using 2-tailed independent-samples *t* tests or Mann-Whitney *U* tests for continuous variables, and chi-square tests or Fisher exact tests for categorical variables, as appropriate. For sST2 comparisons between groups, adjusted analyses were performed using analysis of covariance controlling for confounders. In addition, baseline characteristics were compared between the MACE and non-MACE groups using the 2-tailed independent-samples *t* test for continuous variables and the chi-square test or Fisher exact test, or Fisher Freeman Halton exact test for categorical variables, as appropriate. Crude associations with MACE were explored using univariable logistic regression, and crude odds ratios (ORs) with 95% CIs were reported. Candidate predictors for multivariable modeling were chosen based on clinical plausibility together with univariable screening, with preference given to variables with *P*<.20 while avoiding the redundant representation of the same biological domain and limiting model complexity in view of the available number of events. A 2-sided *P* value <.05 was considered statistically significant for the final multivariable analyses [18,19]. Model development and reporting were guided by transparent prediction-model reporting principles, including TRIPOD (Transparent Reporting of a Multivariable Prediction Model for Individual Prognosis or Diagnosis) and TRIPOD+AI (Transparent Reporting of a multivariable prediction model for Individual Prognosis or Diagnosis plus Artificial Intelligence) recommendations [20,21]. With 47 events, parsimonious models were limited to 3 to 4 predictors, corresponding to approximately 15.7 to 11.8 events per variable, which is consistent with conventional events per variable guidance for logistic regression [20,21]. Sample size adequacy for prediction-model development was additionally assessed using the pmsampsize framework for binary outcomes proposed by Riley et al [22].

To reduce the risk of overfitting, model development followed a prespecified parsimonious candidate-model strategy rather than unrestricted stepwise selection. All candidate models were fitted on the same complete-case dataset to ensure a fair comparison of model performance. There were no missing data for the variables included in the candidate prediction models. All 218 patients had available sST2 measurements, baseline clinical and laboratory variables, and 30-day outcome status. Based on the clinically prioritized core predictors age and sST2, 11 prespecified logistic regression models were examined. Final model selection was based on a joint assessment of optimism-corrected discrimination, calibration, overall fit, parsimony, and clinical interpretability. When candidate models showed comparable optimism-corrected discrimination, preference was given to the model with fewer predictors, a more favorable calibration slope, and greater feasibility for conversion into a simple point-based score. Apparent discrimination for each candidate model was quantified with the area under the receiver operating characteristic curve (AUC) using pROC, with 95% CIs estimated by the DeLong method. Overall fit was additionally assessed with the Akaike information criterion (AIC) and Brier score. Although hypertension showed a signal in univariable analysis, categorical variables with sparse subgroup counts or unstable estimates, particularly hypertension and New York Heart Association class, were not prioritized for the prespecified parsimonious multivariable models. This decision was made because some categories contained relatively small numbers of patients, resulting in wide CIs and reduced estimate stability, with the potential to increase model complexity and overfitting without clear incremental predictive value [18].

Shortlisted candidate models were then subjected to internal validation using bootstrap resampling with the rms package. Apparent and optimism-corrected performance were evaluated with 1000 bootstrap resamples, focusing on AUC, Brier score, calibration intercept, and calibration slope. The final model was selected according to the prespecified rule described above, with priority given to comparable optimism-corrected discrimination, calibration slope, parsimony, and clinical interpretability. The final coefficients and adjusted ORs were then estimated using standard multivariable logistic regression with the glm function under a binomial link. The assessment of calibration was emphasized because adequate calibration is essential for the clinical usefulness of risk prediction models [18,19].

A sensitivity analysis was performed using Firth penalized logistic regression with the logistf package to assess the robustness of coefficient estimates and inference under small-sample conditions and potential sparse-data bias [23,24]. Discrimination of the final logistic model was evaluated by AUC with a 95% CI using pROC. Calibration was examined both numerically and graphically using rms, including the calibration intercept and slope as summary indices and bootstrap-based calibration plots for visual assessment of agreement between predicted and observed risk. Figures were generated with ggplot2; when a smoothed receiver operating characteristic curve was displayed for visual presentation, the reported AUC and 95% CI were still calculated from the original empirical receiver operating characteristic curve.

A simplified point-based score was subsequently derived from the final parsimonious model. First, uniform shrinkage was applied to the regression coefficients using the bootstrap-corrected calibration slope from the final model. The shrunken coefficients were then converted into a pragmatic integer score using clinically interpretable reference values and increments. For each predictor, the shrunken coefficient was first rescaled according to its clinically defined increment: 5 years for age above 60 years, 5 ng/mL for sST2 above 20 ng/mL, and 10 mL/min/1.73 m² for eGFR below 60 mL/min/1.73 m². Let *w_j_* denote the rescaled shrunken coefficient for predictor *j*. Integer points were assigned by dividing each *w_j_* by the smallest absolute rescaled coefficient and rounding to the nearest integer: Points*_j_* = round (*w_j_*/min(|*w*|)). Using this approach, age, sST2, and eGFR were assigned 1, 2, and 1 points, respectively. The total score was obtained by summing the component points across predictors. Negative component points were not allowed. Scores above the maximum observed value in the derivation cohort were not used to generate separate risk estimates and should be interpreted as ≥12 until externally validated. A secondary logistic regression model with total score as the sole predictor was then fitted to translate score values into estimated probabilities of MACE. In this score-only logistic model, the intercept was −2.592, and the coefficient for each 1-point increase in the SAGE-HF score was 0.250, corresponding to an OR of 1.284 per 1-point increase. The predicted probability was calculated as logit(p)=–2.592 + 0.250 × SAGE-HF score. The discrimination of the simplified score was assessed by AUC with 95% CI using pROC, and its calibration was evaluated with Brier score, calibration intercept, calibration slope, and calibration plots using rms [18,19]. Decision curve analysis was performed to evaluate the clinical usefulness of the SAGE-HF score by estimating net benefit across threshold probabilities from 0.05 to 0.50 and comparing the score with treat-all and treat-none strategies [25].

## Results

**Figure 1.**
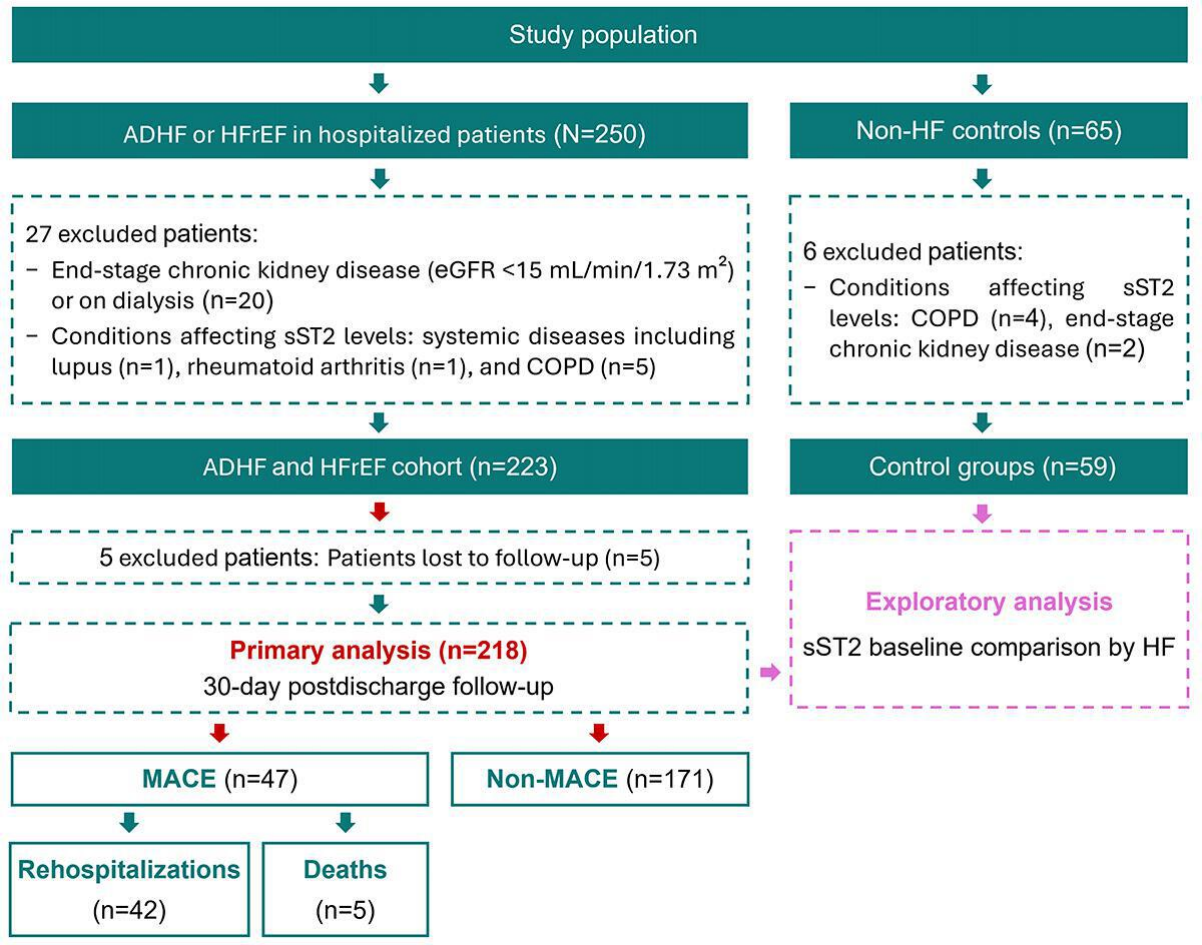
Flow diagram of patients hospitalized with acute decompensated heart failure (ADHF) and/or heart failure with reduced ejection fraction (HFrEF) and non–heart failure (HF) controls. COPD: chronic obstructive pulmonary disease; eGFR: estimated glomerular filtration rate; MACE: major adverse cardiovascular event; sST2: soluble suppression of tumorigenicity 2.

A total of 218 patients with ADHF and/or HFrEF and 59 non-HF controls were included in the final analysis. The participant selection flow is presented in Figure 1. Serum sST2 concentrations were significantly higher in the HF group compared with the non-HF group (median 24.2, IQR 17.0-30.4 ng/mL vs 15.9, IQR 11.7-23.2 ng/mL; *P*<.001). This difference remained statistically significant after adjustment for potential confounders, with both unadjusted and adjusted analyses demonstrating consistent results (Table S1 in Multimedia Appendix 1). Among the 218 patients with HF, 47 patients experienced 30-day postdischarge MACE, including 42 rehospitalizations for HF and 5 deaths. The baseline characteristics of the overall study population and their comparisons between the MACE and non-MACE groups are presented in Table 1. Patients in the MACE group were significantly older than those in the non-MACE group (mean 74.9, SD 13.0 y vs 68.3, SD 12.1 y; *P*=.001). Additionally, patients with MACE had higher median sST2 concentrations (median 27.6, IQR 23.3-30.6 ng/mL vs 22.6, IQR 15.9-30.2 ng/mL; *P*=.006), higher urea levels (median 10.1, IQR 6.4-13.3 mmol/L vs 6.7, IQR 5.0-9.2 mmol/L; *P*=.001), and lower eGFR (median 44.0, IQR 30.7-61.9 mL/min/1.73 m² vs 62.2, IQR 45.5-84.7 mL/min/1.73 m²; *P*<.001). Serum sodium was also lower in the MACE group (median 134.0, IQR 131.6-138.5 mmol/L vs 136.0, IQR 134.0-139.0 mmol/L; *P*=.02)

**Table 1.** Baseline characteristics of the study population overall and stratified by 30-day postdischarge major adverse cardiovascular events (MACE).

Characteristics	MACE (n=47)	Non-MACE (n=171)	Total (n=218)	*P* value
Age (y), mean (SD)	74.9 (13.0)	68.3 (12.1)	69.7 (12.6)	.001^a^
Female, n (%)	18 (38.3)	79 (46.2)	97 (44.5)	.41^b^
BMI (kg/m^2^), median (IQR)	21.3 (19.7-22.5)	22.4 (19.7-23.8)	21.8 (19.7-23.6)	.13^c^
NYHA^d^ classification, n (%)				.18^e^
NYHA II	3 (6.4)	28 (16.4)	31 (14.3)	
NYHA III	20 (42.6)	82 (48.0)	102 (46.8)	
NYHA IV	24 (51.1)	61 (35.7)	85 (39.0)	
Hypertension, n (%)	40 (85.1)	162 (94.7)	202 (92.7)	.03^b^
Diabetes, n (%)	16 (34.0)	67 (39.2)	83 (38.1)	.52^b^
Dyslipidemia, n (%)	16 (34.0)	64 (37.4)	80 (36.7)	.73^b^
Prior myocardial infarction, n (%)	25 (53.2)	88 (51.5)	113 (51.8)	.83^b^
Stroke, n (%)	2 (4.3)	14 (8.2)	16 (7.3)	.36^f^
Atrial fibrillation, n (%)	10 (21.3)	31 (18.1)	41 (18.8)	.63^b^
Smoking, n (%)	11 (23.4)	47 (27.5)	58 (26.6)	.58^b^
Drinking, n (%)	15 (31.9)	63 (36.8)	78 (35.8)	.53^b^
LVEF^g^ (%), median (IQR)	39.0 (32.5-56.5)	36.0 (29.5-45.0)	36.0 (30.0-48.8)	.17^c^
HF^h^ phenotype, n (%)				.32^e^
HFrEF^i^	28 (59.6)	119 (69.6)	147 (67.4)	
HFmrEF^j^	4 (8.5)	15 (8.8)	19 (8.7)	
HFpEF^k^	15 (31.9)	37 (21.6)	52 (23.9)	
ADHF^l^, n (%)	22 (46.8)	57 (33.3)	79 (36.2)	.09^b^
sST2^m^ (ng/mL), median (IQR)	27.6 (23.3-30.6)	22.6 (15.9-30.2)	24.2 (17.0-30.4)	.006^c^
Serum sodium (mmol/L), median (IQR)	134.0 (131.6-138.5)	136.0 (134.0-139.0)	136.0 (133.0-139.0)	.02^c^
Serum urea (mmol/L), median (IQR)	10.1 (6.4-13.3)	6.7 (5.0-9.2)	7.2 (5.0-10.0)	.001^c^
Serum creatinine (μmol/L), median (IQR)	123.0 (78.5-177.0)	94.0 (71.5-124.0)	98.0 (75.0-129.8)	.03^c^
eGFR^n^ (mL/min/1.73 m^2^), median (IQR)	44.0 (30.7-61.9)	62.2 (45.5-84.7)	57.0 (40.9-82.5)	<.001^c^

a Two-tailed independent sample *t* test.

b Chi-square test.

c Mann-Whitney *U* test.

d NYHA: New York Heart Association. No enrolled patients were classified as NYHA class I.

e Fisher-Freeman-Halton exact test.

f Fisher exact test.

g LVEF: left ventricular ejection fraction.

h HF: heart failure.

i HFrEF: heart failure with reduced ejection fraction.

j HFmrEF: heart failure with mildly reduced ejection fraction.

k HFpEF: heart failure with preserved ejection fraction.

l ADHF: acute decompensated heart failure.

m sST2: soluble suppression of tumorigenicity 2.

n eGFR: estimated glomerular filtration rate.

Among the prespecified candidate models, discrimination ranged from an AUC of 0.714 to 0.747. The model including age, sST2, sodium, and eGFR (M8) showed the highest apparent discrimination (AUC 0.747, 95% CI 0.670‐0.824) and the lowest AIC (207) and Brier score (0.145), whereas the model including age, sST2, and eGFR (M3) demonstrated comparable performance (AUC 0.741, 95% CI 0.664‐0.819) with only minimal differences in AIC and Brier score (Table S3 in Multimedia Appendix 1). After bootstrap validation with 1000 resamples, the M3 model showed stable performance, with an optimism-corrected AUC of 0.725, a Brier score of 0.155, a calibration intercept of 0.009, and a calibration slope of 0.930. Although M8 had slightly better apparent performance, its optimism-corrected AUC was lower than that of M3 (0.722 vs 0.725), and its calibration slope was less favorable (0.881 vs 0.930; Table S4 in Multimedia Appendix 1). Given the minimal incremental gain in discrimination and overall fit with M8, the M3 model was selected as the final model to preserve parsimony. This choice reduced the number of predictors while maintaining comparable predictive performance, supporting model stability in the context of a limited number of events and aligning with recommended events-per-variable considerations. In addition, the exclusion of sodium avoided potential redundancy with other renal and hemodynamic markers, further enhancing model interpretability and clinical applicability. In the final multivariable logistic regression model (Table 2), age (OR 1.04 per year, 95% CI 1.01‐1.07; *P*=.02), sST2 (OR 1.06 per unit, 95% CI 1.01‐1.11; *P*=.01), and eGFR (OR 0.98 per unit, 95% CI 0.97‐1.00; *P*=.02) were independently associated with 30-day MACE. Firth penalized logistic regression yielded similar results, confirming the robustness of the associations for age (OR 1.036, 95% CI 1.01‐1.07; *P*=.02), sST2 (OR 1.055, 95% CI 1.01‐1.10; *P*=.01), and eGFR (OR 0.982, 95% CI 0.97‐1.00; *P=*.02; Table 3).

**Figure 2.**
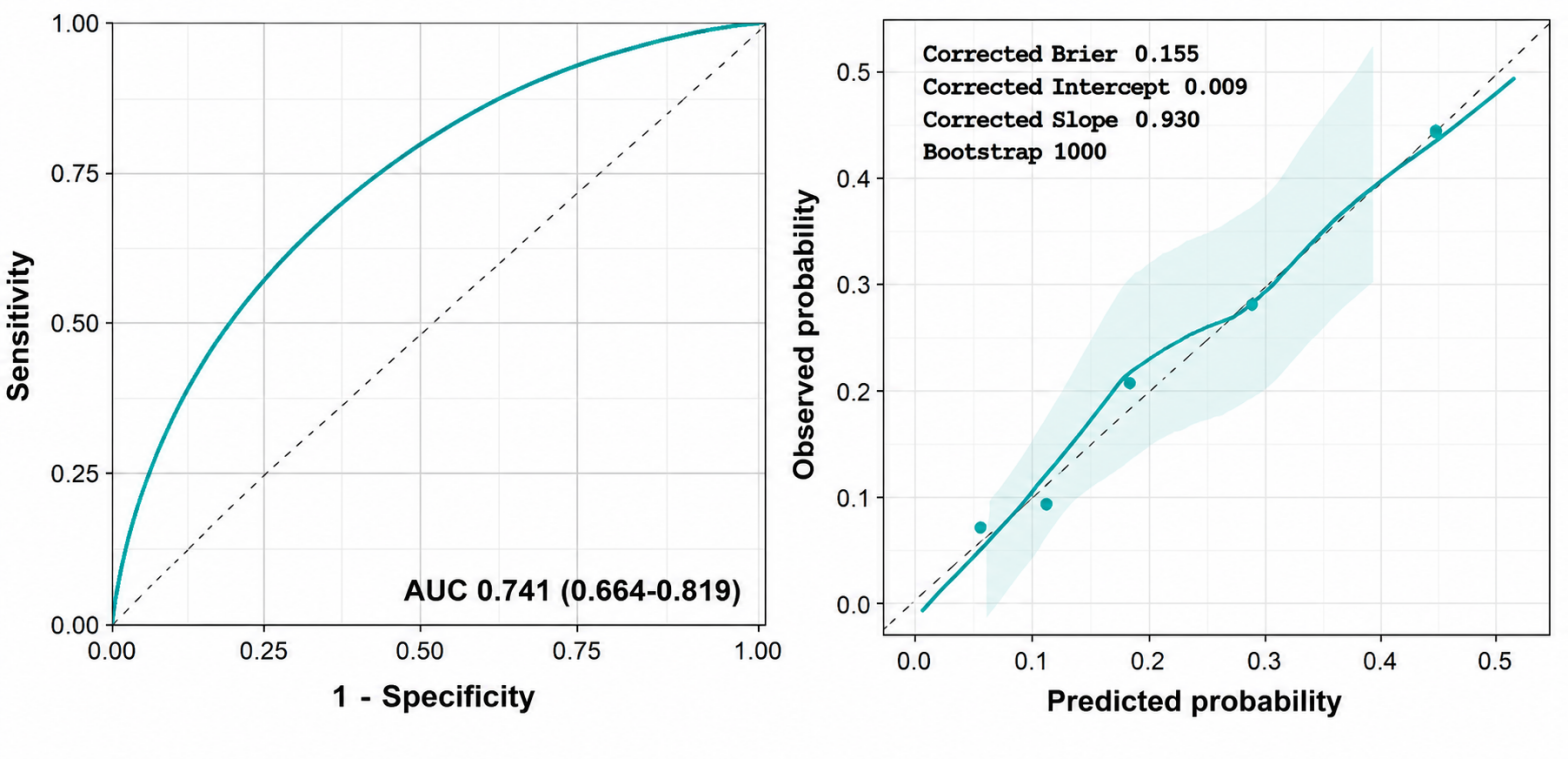
Performance of final multivariable logistic regression model. AUC: area under the receiver operating characteristic curve.

The final multivariable model demonstrated acceptable discrimination (AUC=0.741, 95% CI 0.664‐0.819) and calibration for predicting 30-day MACE (Figure 2).

**Table 2.** Final multivariable logistic regression model for predicting major adverse cardiovascular events (MACE).

Predictor	Unit of comparison	Coefficient	Adjusted OR^a^ (95% CI)	*P* value
Age	+1 year	0.036	1.04 (1.01‐1.07)	.02
sST2^b^	+1 ng/mL	0.055	1.06 (1.01‐1.11)	.01
eGFR^c^	+1 mL/min/1.73 m²	−0.018	0.98 (0.97‐1.00)	.02

a OR: odds ratio.

b sST2: soluble suppression of tumorigenicity 2.

c eGFR: estimated glomerular filtration rate.

**Table 3.** Firth penalized logistic regression sensitivity analysis.

Predictor	Unit of comparison	Coefficient	Adjusted OR^a^ (95% CI)	*P* value
Age	+1 year	0.035	1.036 (1.01‐1.07)	.02
sST2^b^	+1 ng/mL	0.054	1.055 (1.01‐1.10)	.01
eGFR^c^	+1 mL/min/1.73 m²	−0.018	0.982 (0.97‐1.00)	.02

a OR: odds ratio.

b sST2: soluble suppression of tumorigenicity 2.

c eGFR: estimated glomerular filtration rate.

**Figure 3.**
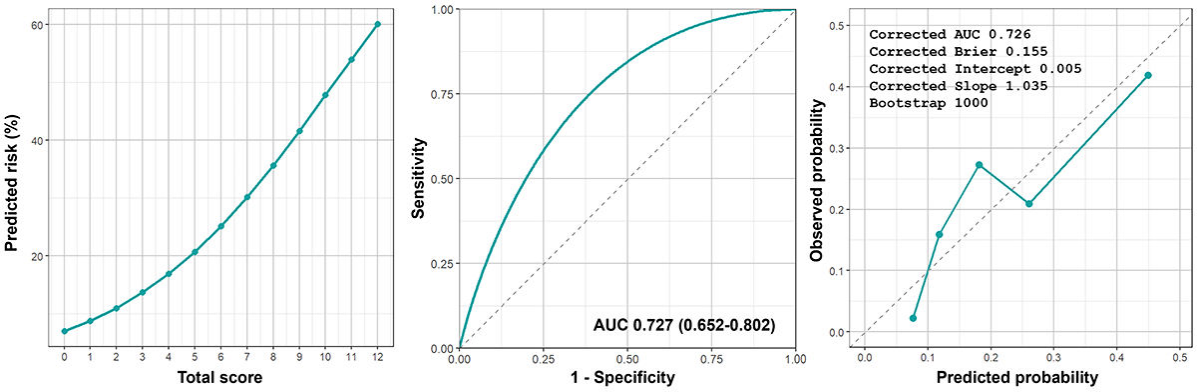
Performance of the simplified risk score for predicting major adverse cardiovascular events (MACE). AUC: area under the receiver operating characteristic curve.

A simplified point-based score was derived from the final model (Table 4). Age was assigned 1 point per 5-year increment above 60 years, sST2 was assigned 2 points per 5 ng/mL increment above 20 ng/mL, and eGFR was assigned 1 point per 10-unit decrease below 60 mL/min/1.73 m². For each component, values below the reference value for age and sST2, or above the reference value for eGFR, were assigned 0 points, and negative points were not allowed. In the derivation cohort, the observed total SAGE-HF score ranged from 0 to 12. Because no patient had a total score above 12 in this cohort, predicted probabilities were tabulated only across the observed score range. For future application, scores above 12 should be categorized as ≥12 and interpreted cautiously because risk estimates beyond the observed range would represent extrapolation and require external validation. The predicted probability of 30-day MACE increased progressively with higher total scores, ranging from 7.0% at a score of 0 to 60.1% at a score of 12 (Table 5). For clinical interpretability, provisional risk strata were defined as low risk (<10%), intermediate risk (10%‐30%), and high risk (>30%) according to predicted 30-day MACE probability. These strata corresponded to SAGE-HF scores of 0 to 1, 2 to 6, and 7 to 12, respectively. The observed MACE risk increased across these strata, from 2.2% in the low-risk group to 22.0% in the intermediate-risk group and 37.0% in the high-risk group. The corresponding exact binomial 95% CIs were 0.1% to 11.5%, 14.9% to 30.6%, and 24.3% to 51.3%, respectively (Table S5 in Multimedia Appendix 1). The simplified score also demonstrated acceptable discrimination and calibration for predicting 30-day MACE (Figure 3). Decision curve analysis suggested that the SAGE-HF score provided greater net benefit than the treat-none strategy and a more favorable net benefit than the treat-all strategy across much of the clinically relevant threshold-probability range, with the advantage over treat-all becoming more evident above approximately the 10% threshold (Figure S1 in Multimedia Appendix 1). In the exploratory benchmark analysis, the SAGE-HF simple score and the adjusted OPTIMIZE-HF in-hospital-derived score showed comparable performance for predicting 30-day postdischarge MACE. The SAGE-HF simple score had an apparent AUC of 0.727, bootstrap 95% CI 0.656‐0.796, and bootstrap-corrected AUC of 0.726. The adjusted OPTIMIZE-HF-derived score had an apparent AUC of 0.731, bootstrap 95% CI of 0.659‐0.805, and bootstrap-corrected AUC of 0.730. The difference in AUC between the 2 scores was minimal and not statistically significant (Tables S6 and S7 in Multimedia Appendix 1).

**Table 4.** Simplified point-based score derived from the final parsimonious model^a^.

Predictor	Reference value	Increment	Assigned points
Age	60 years	+5 years	+1
sST2^b^	20 ng/mL	+5 ng/mL	+2
eGFR^c^	60 mL/min/1.73 m²	−10 mL/min/1.73 m²	+1

a Points were assigned by rounding the ratio of each clinically rescaled shrunken coefficient to the smallest rescaled coefficient.

b sST2: soluble suppression of tumorigenicity 2.

c eGFR: estimated glomerular filtration rate.

**Table 5.** Predicted probability of major adverse cardiovascular events (MACE) according to the total simplified risk score (SAGE-HF^a^ score).

Total score	Predicted risk, % (95% CI)
0	7.0 (3.5‐13.2)
1	8.8 (4.9‐15.2)
2	11.0 (6.8‐17.4)
3	13.7 (9.1‐20.0)
4	16.9 (12.1‐23.2)
5	20.7 (15.5‐27.2)
6	25.2 (19.3‐32.1)
7	30.2 (23.1‐38.4)
8	35.7 (26.8‐45.6)
9	41.6 (30.5‐53.6)
10	47.8 (34.2‐61.7)
11	54.0 (37.9‐69.3)
12	60.1 (41.7‐76.1)

a SAGE-HF: soluble ST2, age, and estimated glomerular filtration rate–heart failure.

## Discussion

### Principal Findings

In this two-center prospective study, serum sST2 concentrations were significantly higher in patients with HF compared with non-HF controls. In the two-center prospective HF cohort, 21.6% (47/218) of the patients experienced 30-day postdischarge MACE, comprising 19.3% (42/218) HF rehospitalizations and 2.3% (5/218) deaths. Although hypertension was less frequent in the MACE group, this crude inverse association should be interpreted cautiously and should not be considered evidence of a protective effect. Hypertension was recorded as a binary history-based comorbidity and may not reflect hemodynamic status during the index hospitalization. This finding may reflect residual confounding, selection or collider bias, the high overall prevalence of hypertension in the cohort, and the small number of patients without hypertension. In a sensitivity analysis, admission systolic blood pressure was not associated with 30-day MACE (OR 0.99 per mm Hg, 95% CI 0.985‐1.013; *P*=.89), supporting the interpretation that the inverse association for categorical hypertension was unlikely to represent a true protective effect. Patients with events were older and had higher admission sST2 and worse renal indices. Among prespecified candidate models, a parsimonious model including age, sST2, and eGFR achieved acceptable discrimination (AUC 0.741) and favorable internal validation with good calibration characteristics. A simplified integer score (SAGE-HF score) derived from the final model produced a clinically interpretable risk gradient, with predicted event probabilities rising from low single digits to approximately 60% across the observed score spectrum.

Age, renal function, and sST2 each contributed distinct prognostic information in this cohort. Patients with 30-day MACE were older than those without MACE (mean 74.9, SD 13.0 y vs 68.3, SD 12.1 y), and age remained independently associated with the end point, with an adjusted OR of 1.04 (95% CI 1.01‐1.07) per year. This finding is consistent with the recognized vulnerability of older adults with HF, who often have greater comorbidity burden, frailty, and reduced physiologic reserve [26]. Renal dysfunction was also more prominent in patients with events. The MACE group had lower eGFR than the non-MACE group (median 44.0, IQR 30.78-61.94 mL/min/1.73 m² vs 62.24, IQR 45.53-84.77 mL/min/1.73 m²), and eGFR remained independently associated with MACE, with an adjusted OR of 0.98 (95% CI 0.97‐1.00) per 1 mL/min/1.73 m². This supports the clinical relevance of cardiorenal interaction in early postdischarge risk after HF hospitalization [27]. Admission sST2 was higher in patients who developed MACE than in those who did not (median 27.61, IQR 23.36-30.62 ng/mL vs 22.68, IQR 15.94-30.27 ng/mL) and remained independently associated with 30-day MACE after adjustment for age and eGFR, with an adjusted OR of 1.06 (95% CI 1.01‐1.11) per ng/mL. This finding is consistent with prior literature linking higher sST2 concentrations to adverse outcomes in acute and chronic HF settings [9,28]. Mechanistically, sST2 reflects the activation of the IL-33/ST2 pathway and may capture myocardial stress, inflammation, and profibrotic remodeling [8,29]. In this cohort, the contribution of sST2 was observed despite the inclusion of renal function in the model, supporting its potential value as a complementary biomarker in short-term risk stratification.

A key finding of this study is that a 3-predictor model incorporating age, admission sST2, and eGFR provided clinically interpretable short-term risk stratification in a cohort with 47 MACE among 218 hospitalized patients with HF. The final model achieved an apparent AUC of 0.741 (95% CI 0.664‐0.819), and its performance remained stable after internal validation with 1000 bootstrap resamples, with an optimism-corrected AUC of 0.725, Brier score of 0.155, calibration intercept of 0.009, and calibration slope of 0.930. These values indicate acceptable discrimination and calibration for an early postdischarge outcome, while also showing the expected degree of optimism in a relatively small derivation cohort. Compared with the 4-predictor model including serum sodium, the 3-predictor model had comparable or better optimism-corrected discrimination and a more favorable calibration slope, supporting the decision to prioritize parsimony.

The simplified SAGE-HF score further translated this model into a bedside format. In the derivation cohort, the predicted 30-day MACE probability increased from 7.0% at a score of 0 to 60.1% at a score of 12. When provisional risk strata were applied, the observed MACE risk was 2.2% in the low-risk group, 22.0% in the intermediate-risk group, and 37.0% in the high-risk group, with exact binomial 95% CIs of 0.1% to 11.5%, 14.9% to 30.6%, and 24.3% to 51.3%, respectively. This gradient is clinically relevant because the overall 30-day MACE rate was 21.6%, comprising 42 HF rehospitalizations and 5 deaths. However, because the low-risk stratum included only 1 event and its upper confidence limit exceeded 10%, the proposed <10% threshold should be interpreted as provisional rather than definitive.

These findings should be interpreted in the context of previous HF prediction literature. Prior studies have emphasized that 30-day readmission after HF hospitalization is difficult to predict because early events are driven by heterogeneous clinical and health-system factors [4]. Machine learning and traditional models for 30-day HF readmission have also shown variable performance and limited external validation across settings [30]. Against this background, the SAGE-HF model does not eliminate the need for clinical judgment, but it offers a low-burden and biologically interpretable approach using 3 readily available predictors. The inclusion of sST2 is consistent with literature showing that sST2 reflects myocardial stress, inflammation, fibrosis, and remodeling and has prognostic value in HF [28]. In this cohort, sST2 remained associated with MACE after adjustment for age and eGFR, supporting its potential role as a complementary biomarker rather than a replacement for standard clinical assessment.

From a postdischarge care perspective, the practical value of the SAGE-HF score lies in its ability to identify patients who may need more intensive early follow-up. This potential clinical usefulness was also supported by decision curve analysis, which showed a favorable net benefit of the SAGE-HF score across a clinically relevant range of threshold probabilities. Timely postdischarge care and structured transition-of-care strategies have been emphasized in HF management and have been associated with lower readmission rates in HF populations [31,32]. In this cohort, patients with scores ≥7 had an observed 30-day MACE risk of 37.0%, suggesting that this group may be considered for closer follow-up, medication optimization, early reassessment, or structured telephone monitoring after discharge. However, these risk categories remain exploratory and should not be used as definitive clinical thresholds until validated in larger and geographically diverse cohorts.

### Limitations

This study has several strengths. The prospective design and inclusion of patients from 2 different centers enhance the reliability of the findings. The model was developed based on a prespecified and transparent strategy, with internal validation using bootstrap methods and comprehensive assessment of calibration. In addition, the inclusion of a non-HF comparison group allowed for an exploratory comparison of sST2 levels between patients with HF and individuals without HF. A Firth penalized logistic regression analysis was also performed to assess the robustness of the model and minimize potential bias related to the limited number of events. Furthermore, a simplified risk score was derived to facilitate clinical application, particularly in discharge planning and early postdischarge risk stratification. However, several limitations should be considered. First, the number of events was relatively small, which limited the number of predictors that could be included and may affect the stability of the model. To contextualize sample size adequacy for prediction-model development, an additional assessment using the pmsampsize framework was performed (Table S8 in Multimedia Appendix 1). With an anticipated C-statistic of 0.74, an outcome prevalence of 0.216, and 15 candidate predictor parameters before model reduction, the estimated minimum sample size was 1031 patients, corresponding to 223 events. In a sensitivity calculation using 19 candidate predictor parameters, the estimated requirement increased to 1306 patients, corresponding to 283 events. Because the present derivation cohort included 218 patients and forty-seven 30-day MACE, the study was underpowered for fitting a full multivariable prediction model containing all candidate predictors before reduction. Therefore, this study should be interpreted as an internally validated derivation study of a deliberately parsimonious score rather than a fully powered development study for a large multivariable prediction model. Second, the follow-up period was limited to 30 days, which may not fully reflect longer-term outcomes after HF hospitalization. In addition, death before rehospitalization may act as a competing event when rehospitalization is analyzed separately. Because the dataset recorded the occurrence of 30-day MACE rather than complete component-specific time-to-event data, a Fine-Gray competing-risk analysis could not be performed appropriately. Third, short-term outcomes after discharge may also be influenced by health care system factors, such as access to follow-up, medication availability, and care coordination, which were not fully captured in this study. In addition, the counterintuitive inverse association between recorded hypertension and 30-day MACE may reflect residual confounding, selection or collider bias, and the limitation of using a history-based binary comorbidity variable rather than detailed longitudinal blood pressure, treatment, or hemodynamic data. Fourth, serum sST2 was measured using a research-use Elabscience ELISA kit rather than the US Food and Drug Administration–cleared Presage ST2 Assay. Therefore, absolute sST2 concentrations in this study should not be directly compared with Presage-based cohorts, and the Presage 35 ng/mL cutoff is not directly transferable. The observed associations and the SAGE-HF score should be interpreted as assay-specific findings that require external validation, ideally including assay harmonization or comparison with clinically validated sST2 platforms. Fifth, the non-HF comparison group was not formally matched to the HF cohort by age, sex, or BMI. Therefore, comparisons between patients with HF and non-HF comparison participants should be interpreted as exploratory and were not used in the development, validation, or performance assessment of the SAGE-HF score. Sixth, although this study included patients from 2 hospitals, the number of centers was small and the findings may not be generalizable to other regions, hospital levels, or HF populations with different etiology patterns. Center-level influence was assessed using a fixed-effect sensitivity analysis, but external validation in larger and geographically diverse multicenter cohorts remains necessary. In addition, formal comparison with some established HF risk scores was limited because several required predictors for these tools, particularly the OPTIMIZE-HF postdischarge mortality score, were not systematically collected in this cohort. Finally, the proposed risk thresholds are exploratory and were derived from the current cohort. They should not be used as definitive clinical decision thresholds before external validation. In addition, the low-risk stratum contained only 1 MACE. Although the observed event rate was low, the exact 95% CI extended above 10%, indicating that the proposed low-risk threshold requires confirmation in larger external validation cohorts. Further studies are needed to validate these findings in larger and more diverse populations, with longer follow-up and adequate event numbers. Such work may help clarify the role of this model in routine clinical practice.

### Conclusions

In this two-center prospective cohort of patients hospitalized for HF, 21.6% (47/218) experienced 30-day postdischarge MACE. Admission age, sST2, and eGFR were independently associated with risk and were integrated into the SAGE-HF score, which demonstrated acceptable predictive performance. The score provided clear risk stratification with a graded increase in predicted MACE probability. External validation is warranted before routine clinical application.

## Supplementary material

10.2196/97879 Multimedia Appendix 1 Supplementary tables and figure presenting baseline comparisons, univariable predictors, prediction model performance, internal validation, risk stratification, benchmark comparison, sample size assessment, and decision curve analysis for the soluble ST2, age, and estimated glomerular filtration rate–heart failure score.

## Abbreviations

ADHF: acute decompensated heart failure
AIC: Akaike information criterion
AUC: area under the receiver operating characteristic curve
eGFR: estimated glomerular filtration rate
ELISA: enzyme-linked immunosorbent assay
HF: heart failure
HFmrEF: heart failure with mildly reduced ejection fraction
HFpEF: heart failure with preserved ejection fraction
HFrEF: heart failure with reduced ejection fraction
IL: interleukin
LVEF: left ventricular ejection fraction
MACE: major adverse cardiovascular events
OR: odds ratio
SAGE-HF: soluble ST2, age, and estimated glomerular filtration rate–heart failure
sST2: soluble suppression of tumorigenicity 2
TRIPOD: Transparent Reporting of a Multivariable Prediction Model for Individual Prognosis or Diagnosis
TRIPOD+AI: Transparent Reporting of a Multivariable Prediction Model for Individual Prognosis or Diagnosis plus Artificial Intelligence
