# Supplementary material for: Development and Internal Validation of the Soluble ST2, Age, and Estimated Glomerular Filtration Rate–Heart Failure Score for Predicting 30-Day Major Adverse Cardiovascular Events After Heart Failure Hospitalization in a Two-Center Vietnamese Cohort: Prospective Cohort Study
Source: JMIR Cardio. 2026 Jul 29;10:e97879. doi: 10.2196/97879 (PMC13418553; doi:10.2196/97879)
Supplement: Multimedia Appendix 1 [file cardio-v10-e97879-s001.docx]

**Supplementary Table 1.** Comparison of baseline characteristics and serum sST2 concentrations between patients with HF and non-HF controls

| **Characteristics** | **HF**  **(n = 218)** | **Non-HF controls**  **(n= 59)** | ***P*** | |
| --- | --- | --- | --- | --- |
| Age (years), mean (SD) | 69.7 (12.6) | 64 (12.5) | .005^a^ | |
| Female | 97 (44.5) | 43 (72.9) | <.001^b^ | |
| BMI (kg/m^2^), median (IQR) | 21.8 (3.8) | 24.6 (4.9) | <.001^c^ | |
| Hypertension | 202 (92.7) | 56 (94.9) | .39^d^ | |
| Diabetes | 83 (38.1) | 15 (25.4) | .07^b^ | |
| Dyslipidemia | 80 (36.7) | 8 (13.6) | <.001^b^ | |
| Stroke | 16 (7.3) | 7 (11.9) | .26^b^ | |
| Atrial fibrillation | 41 (18.8) | 1 (1.7) | .002^b^ | |
| Smoking | 58 (26.6) | 8 (14.0) | .04^b^ | |
| Alcohol drinking | 78 (35.8) | 14 (26.4) | .08^b^ | |
| sST2 concentration (ng/mL), median (IQR) | 24.2 (13.4) | 15.9 (11.5) | <.001^c^* | |
| ^a^Independent sample T test, ^b^Chi-square test, ^c^Mann-Whitney U test, ^d^Fisher’s exact test  *Both unadjusted and adjusted p values (from ANCOVA controlling for age, sex, BMI, atrial fibrillation, diabetes mellitus, dyslipidemia, smoking, and drinking) | | | |  |

**Supplementary Table 2.** Univariable logistic regression analyses for predictors of 30-day post-discharge MACE

| **Characteristics** | **Crude OR** | **95% CI** | ***P*** |
| --- | --- | --- | --- |
| Age, per 1 year | 1.05 | 1.018–1.078 | **.001** |
| sST2, per 1 unit | 1.06 | 1.019–1.109 | **.005** |
| BMI, per 1 kg/m² | 0.91 | 0.825–1.009 | .09 |
| Urea on admission, per 1 mmol/L | 1.10 | 1.032–1.183 | **.004** |
| eGFR, per 1 ml/min/1.73 m^2^ | 0.97 | 0.960–0.988 | **<.001** |
| Serum sodium on admission, per 1 mmol/L | 0.93 | 0.877–0.998 | **.04** |
| Diabetes mellitus | 0.80 | 0.407–1.577 | .52 |
| Dyslipidemia | 0.86 | 0.438–1.700 | .67 |
| Acute decompensated heart failure | 1.76 | 0.910–3.394 | .09 |
| Female | 0.72 | 0.368–1.389 | .34 |
| Alcohol use | 0.80 | 0.404–1.598 | .53 |
| Hypertension | 0.31 | 0.111–0.904 | **.03** |
| LVEF, per 1% | 1.02 | 0.995–1.043 | .13 |
| Heart rate, per 1 beat/min | 1.01 | 0.994–1.026 | .24 |
| Previous myocardial infarction | 1.07 | 0.561–2.046 | .83 |
| Smoking | 0.81 | 0.381–1.717 | .58 |
| Stroke | 0.50 | 0.114–2.280 | .37 |
| Atrial fibrillation | 1.22 | 0.549–2.715 | .63 |
| Systolic blood pressure on admission | 0.99 | 0.985–1.013 | .89 |

**Supplementary Table 3.** Performance of prespecified parsimonious candidate models for predicting MACE

| **Model** | **AUC** | **95% CI** | **AIC** | **Brier score** |
| --- | --- | --- | --- | --- |
| Age + sST2 + ADHF (M1) | 0.716 | 0.636–0.795 | 215 | 0.154 |
| Age + sST2 + Sodium (M2) | 0.719 | 0.640–0.797 | 212 | 0.151 |
| Age + sST2 + eGFR (M3) | 0.741 | 0.664–0.819 | 209 | 0.149 |
| Age + sST2 + Urea (M4) | 0.737 | 0.660–0.814 | 211 | 0.151 |
| Age + sST2 + ADHF + Sodium (M5) | 0.719 | 0.640–0.797 | 214 | 0.151 |
| Age + sST2 + ADHF + eGFR (M6) | 0.741 | 0.662–0.819 | 210 | 0.147 |
| Age + sST2 + ADHF + Urea (M7) | 0.739 | 0.661–0.817 | 213 | 0.150 |
| Age + sST2 + Sodium + eGFR (M8) | 0.747 | 0.670–0.824 | 207 | 0.145 |
| Age + sST2 + Sodium + Urea (M9) | 0.745 | 0.669–0.822 | 210 | 0.147 |
| Age + sST2 + BMI (M10) | 0.725 | 0.646–0.803 | 213 | 0.153 |
| Age + sST2 + LVEF (M11) | 0.714 | 0.635–0.792 | 215 | 0.154 |

**Supplementary Table 4.** Apparent and bootstrap-corrected performance of prespecified parsimonious candidate models

| **Model** | **Apparent performance** | | | | **Optimism-corrected performance** | | | |
| --- | --- | --- | --- | --- | --- | --- | --- | --- |
|  | **AUC** | **Brier score** | **Calibration intercept** | **Calibration slope** | **AUC** | **Brier score** | **Calibration intercept** | **Calibration slope** |
| M3 | 0.741 | 0.149 | 0.000 | 1.000 | 0.725 | 0.155 | 0.009 | 0.930 |
| M8 | 0.747 | 0.145 | 0.000 | 1.000 | 0.722 | 0.154 | 0.004 | 0.881 |
| M4 | 0.737 | 0.151 | 0.000 | 1.000 | 0.721 | 0.157 | 0.002 | 0.925 |

**Supplementary Table 5.** Provisional risk strata according to predicted 30-day MACE risk using the SAGE-HF score

| **Risk stratum** | **Predicted risk threshold** | **SAGE-HF score range** | **n** | **MACE events** | **Exact**  **95%CI** | **Mean predicted risk, %** | **Median score** |
| --- | --- | --- | --- | --- | --- | --- | --- |
| Low risk | <10% | 0–1 | 46 | 1 (2.2) | 0.1 – 11.5 | 7.6 | 0 |
| Intermediate risk | 10%–30% | 2–6 | 118 | 26 (22.0) | 14.9 – 30.6 | 17.4 | 4 |
| High risk | >30% | 7–12 | 54 | 20 (37.0) | 24.3 – 51.3 | 42.5 | 9 |

Exact 95% confidence intervals were calculated using the exact binomial method. Risk strata were defined according to predicted 30-day MACE probability and should be interpreted as provisional pending external validation.

**Supplementary Table 6. Exploratory benchmark comparison between the SAGE-HF simple score and an adjusted OPTIMIZE-HF**

| **Score** | **n** | **Apparent AUC** | **Bootstrap 95% CI** | **Bootstrap-corrected AUC** | **Brier score** | **Bootstrap-corrected Brier** | **Bootstrap-corrected calibration intercept** | **Calibration slope** |
| --- | --- | --- | --- | --- | --- | --- | --- | --- |
| **SAGE-HF simple score** | 218 | 0.727 | 0.656–0.796 | 0.726 | 0.152 | 0.155 | 0.005 | 1.035 |
| **OPTIMIZE-HF in-hospital-derived score** | 218 | 0.731 | 0.659–0.805 | 0.730 | 0.156 | 0.160 | -0.002 | 1.060 |

**Supplementary Table 7. Observed 30-day post-discharge MACE according to SAGE-HF and adjusted OPTIMIZE-HF-derived risk strata**

| **Score** | **Risk group** | **n** | **MACE, n (%)** |
| --- | --- | --- | --- |
| SAGE-HF | Low, 0–1 | 46 | 1 (2.2) |
| SAGE-HF | Intermediate, 2–6 | 118 | 26 (22.0) |
| SAGE-HF | High, ≥7 | 54 | 20 (37.0) |
| OPTIMIZE-HF in-hospital-derived | Lower tertile | 87 | 5 (5.7) |
| OPTIMIZE-HF in-hospital-derived | Middle tertile | 78 | 23 (29.5) |
| OPTIMIZE-HF in-hospital-derived | Higher tertile | 53 | 19 (35.8) |

**Supplementary Table 8.** Sample size assessment for prediction-model development using the pmsampsize R package

| **Anticipated C-statistic** | **Outcome prevalence** | **Candidate predictor parameters** | **Required sample size** | **Required events** | **Events per predictor parameter** |
| --- | --- | --- | --- | --- | --- |
| 0.74 | 0.216 | 15 | 1031 | 223 | 14.85 |
| 0.74 | 0.216 | 19 | 1306 | 283 | 14.85 |
| 0.741 - Current apparent AUC | 0.216 | Final model: 3 predictors | 218 | 47 | 15.67 |


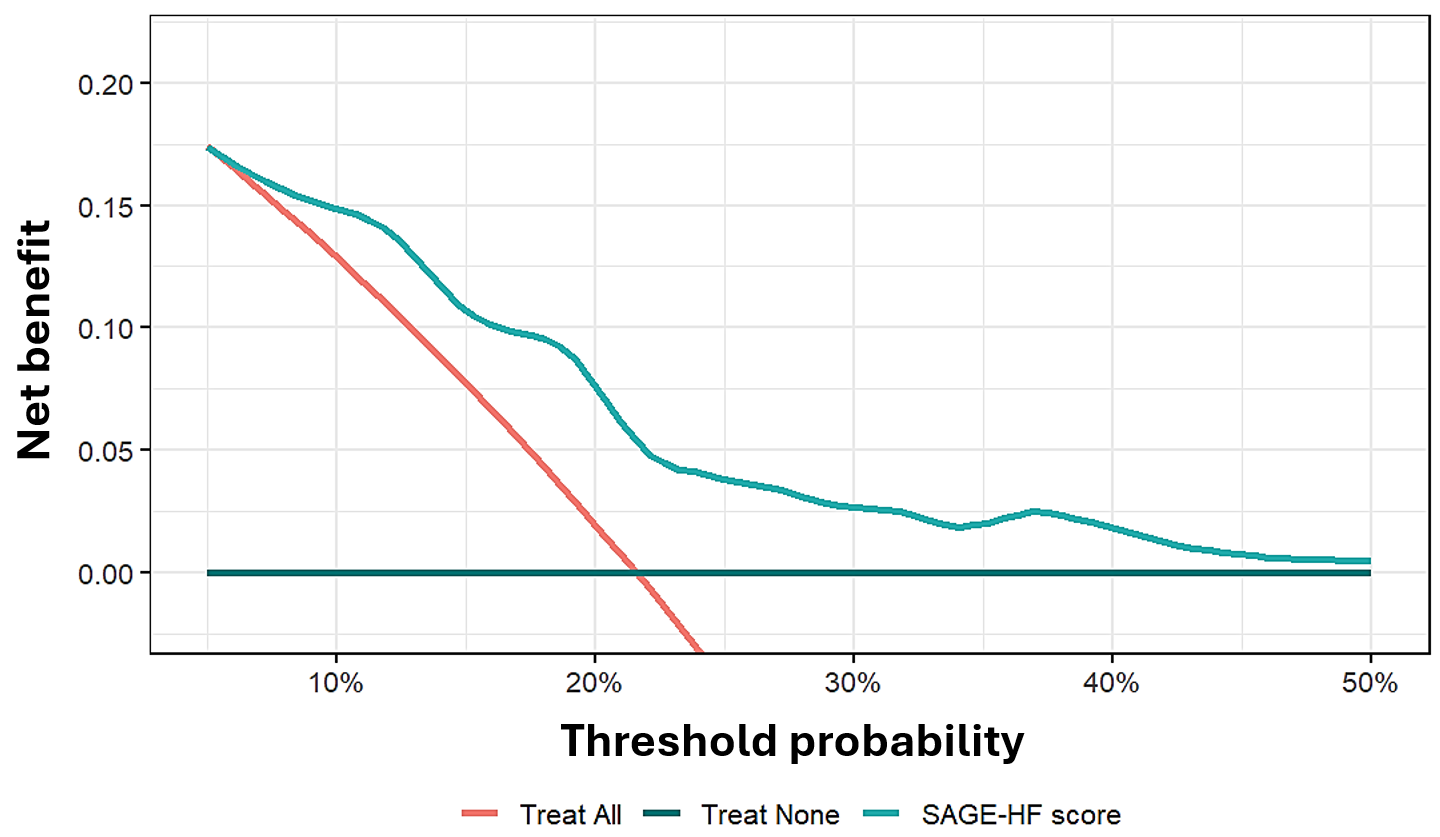


**Supplementary Figure 1.** Decision curve analysis of the SAGE-HF score
